# Application of tergitol in liver decellularization and bioink preparation based on the obtained extracellular matrix

**DOI:** 10.3389/fbioe.2025.1680938

**Published:** 2025-10-23

**Authors:** Paweł Mirosław, Katarzyna Kosowska, Wiktoria Serafin, Radosław Piwowar, Agnieszka Romanik-Chruścielewska, Kinga Westphal, Michał Wszoła, Marta Klak

**Affiliations:** ^1^ Foundation of Research and Science Development, Warsaw, Poland; ^2^ Polbionica Ltd., Warsaw, Poland; ^3^ Medispace Medical Centre, Warsaw, Poland

**Keywords:** liver, decellularization, extracellular matrix, detergents, bioink

## Abstract

Biological materials derived from decellularized tissues are of great interest in the field of tissue engineering. Artificial organoids can be used in drug discovery or toxicological studies, potentially reducing the reliance on animal models. They also serve as valuable models for cancer and virology research. In the study, 13 slightly different detergent-enzymatic decellularization processes were performed. The end products of each process were evaluated for their DNA content, residual lipids, sulfated glycosaminoglycans (sGAG), collagen and detergent residues. To our knowledge, for the first time, Tergitol was used in liver decellularization. A method for detecting residual Tergitol content in the matrix was also developed. A significant reduction in DNA and fat content was achieved, as well as preservation of collagen. However, a marked loss of sGAGs was observed. The most promising variant involved gradient decellularization using Tergitol and ammonium hydroxide combined with pre-digestion by trypsin, which yielded the most favorable results and showed no cytotoxicity. A bioink was subsequently formulated from the decellularized matrix, which has potential applications in 3D bioprinting of artificial liver tissue.

## 1 Introduction

Liver diseases cause approximately two million deaths annually worldwide. Cirrhosis and liver cancer are the 11th and 16th most common causes of death, respectively, accounting for 3.5% of all deaths (Asrani et al., 2019). To date, liver transplantation is widely considered the only effective therapy for end-stage liver disease (Dutkowski et al., 2011). However, in the face of a global shortage of organs for transplantation (Arulraj and Neuberger, 2011), including the rejection of a large proportion of potential donors (Orman et al., 2013), 20% of patients die while on the waiting list (Dutkowski et al., 2011).

Bioengineering is a promising strategy that could potentially find applications in liver tissue regeneration or recreation of a functional liver substitute. Artificial liver organoids can be used in the drug discovery, toxicology, virology or oncology research which would reduce the use of model animals (McGill and Jaeschke, 2019).

Recent developments in this field have enabled the use of decellularized organs as scaffolds that allow the preservation of the native structure of the extracellular matrix (ECM). Once repopulated with cells, these scaffolds can serve as biomaterials for the development of 3D tissue models (Arulraj and Neuberger, 2011).

ECM is a non-cellular component found in all tissues and organs of the body. In the healthy liver, the ECM comprises <3% of the relative area and is largely restricted to Glisson’s capsule, portal tracts and central veins (Bedossa and Paradis, 2003). It is a complex network of hydrated proteins and polysaccharides, providing physical support for cells and also serves as a reservoir of cytokines, growth factors, bioactive peptides that modulate immune responses. The various signals provided by the ECM are detected by receptors on the cell surface, triggering intracellular signaling cascades affecting processes of morphogenesis, tissue repair, differentiation and homeostasis (McQuitty et al., 2020). In depth proteomic analysis 105 different ECM proteins have been identified that make up the healthy human liver matrix (Naba et al., 2014).

Naturally derived extracellular matrix scaffolds that recreate the native cellular niche can be generated by removing cells from tissues or organs through decellularization. Variety of acellular scaffolds have already been obtained, for example, from: trachea (Dimou et al., 2019), aortic valves (Faggioli et al., 2022), urinary bladder (Kao et al., 2020), human pancreas (Sackett et al., 2018), kidney (Shahraki et al., 2022) and from liver (Maghsoudlou et al., 2016). Decellularization of the whole body of the rat was also demonstrated (Taylor et al., 2021).

Generally, a desirable decellularization process begins with lysis of the cells, dissociation of nuclear and cytoplasmic components from the ECM using: physical, enzymatic, chemical methods or their combination. Physical methods include the use of freeze-thaw cycles, high hydrostatic pressure, sonication or supercritical fluids (Rabbani et al., 2021), but they are not very effective, for example, freezing/thawing alone does not fully remove all cellular components (Burk et al., 2014). The most commonly used tissue decellularization techniques are immersion in detergent and/or enzyme solutions with mechanical agitation (Shahraki et al., 2022; Faggioli et al., 2022) or perfusion (Struecker et al., 2017). Detergents, such as sodium dodecyl sulphate (SDS) and Triton X-100 are the most commonly applied chemical agents for decellularizing procedures. Furthermore Triton X-100 was added to the candidate list by the European Chemical Agency (ECHA) as a substance of concern due to its degradation into a substances with endocrine-disrupting properties (European Chemicals Agency, 2021). In light of this, the search for alternative, less hazardous decellularization agents appears to be of particular importance. Following decellularization, all cell elements and residual chemicals must be thoroughly rinsed from the scaffold especially if it will be used in medical applications. Residual content of detergents remaining in the obtained material may contribute to the atypical phenotype and lower cell viability (White et al., 2017).

“Bioinks” are important tools to produce artificial constructs of living tissues using 3D bioprinting. Bioinks are most often created by incorporating living cells into their composition, so they should be able to imitate the properties of native tissues. One of the components of bioink can be decellularized extracellular matrix (dECM).

An important aspect of developing a new biomaterial is to ensure it possesses the appropriate properties to meet both technical and biological requirements. Attention should be given to material properties including printability, viscosity, degradability, functionality, and biocompatibility. Various protocols effectively remove intracellular components while preserving the native ECM structure, resulting in scaffolds capable of supporting the recellularization process. However, there is little consensus on the optimal method for decellularizing hepatic tissue. The choice of detergents used for decellularization affects both the effectiveness of the process and the compositional quality of the bioengineered construct. Therefore, the selection of appropriate decellularization agents is crucial. Efficiency of decellularization dependent also upon many other factors, including the origin of the tissue and its characteristics, for example, cellularity, density, thickness, lipid content, architecture and composition of ECM (Li et al., 2016). The decellularization process can alter the composition of ECM and thus influence the bioink characteristics (Fernandez-Perez and Ahearne, 2019).

The aim of this study was, firstly, to demonstrate the feasibility of different protocols using various decellularization factors such as detergents (SDS, sodium deoxycholate (SD), Triton X-100, Tergitol 15.S.9), enzymes (trypsin, DNase), and a chelator (EDTA) to generate an acellular natural matrix from porcine liver, and secondly, to assess their effect on the composition of the final product. To our knowledge, Tergitol was used for the first time in liver decellularization. Additionally, we propose a new HPLC-based procedure for the direct quantification of detergent - Tergitol 15.S.9 residues trapped in the scaffold of decellularized powdered livers. A bioink based on the developed dECM was also produced, and its basic properties were characterized, potentially enabling 3D bioprinting of liver tissue models.

## 2 Materials and methods

### 2.1 Decellularization

Porcine livers were collected at local slaughterhouse and frozen at −20 °C. The organs were thawed, manually cleaned, rinsed with betadine solution (2% v/v) and subsequently in streptomycin solution (0.1 mg*ml^-1^) dissolved in phosphate-buffered saline (PBS). The shredded and mixed material was weighed and placed in 2 L bottles. We carried out 13 different decellularization protocols. They are listed in Table 1. Bottles with crushed liver tissue were flooded with selected detergent solution: SDS (Sigma Aldrich, US), SD (Sigma Aldrich, US), Triton X-100 (Sigma Aldrich, US) or Tergitol 15.S.9 (Sigma Aldrich, US) with the addition of ammonium hydroxide (0.1%). Some of them were pre-incubated in a trypsin-EDTA solution (0.05% (w/v) trypsin (Gibco, US) and 0.02% (w/v) EDTA (Sigma Aldrich, US)) before the detergent treatment.

**TABLE 1:** Protocols used for liver decellularization.

Protocol	Incubation in trypsin solution	Detergent/number of rinses	Number of washes with PBS solution
1	-	1% Triton X-100 + NH_4_OH/15	14
2	-	1% SDS/15	14
3	+	1% Triton X-100 + NH_4_OH/15	14
4	+	1% SDS/15	14
5	-	1% Tergitol + NH_4_OH/15	14
6	+	1% Tergitol + NH_4_OH/15	14
7	+	1% SDS/7 1% Triton X-100 + NH_4_OH/5	16
8	+	1% Triton X-100 + NH_4_OH/7 1% SDS/5	16
9	+	1% SDS/6 1% Tergitol + NH_4_OH/6	16
10	+	1% Tergitol + NH_4_OH/6 1% SDS/6	16
11	+	1% SD/6 1% Tergitol + NH_4_OH/6	16
12	+	3% Tergitol + NH_4_OH/4 1% Tergitol + NH_4_OH/4 0.5% Tergitol + NH_4_OH/4	16
13	-	1% SDS/6 1% Tergitol + NH_4_OH/6	16

The solutions with the material were placed in a mixing incubator at 150 r*m^-1^ at 4 °C for 17 h. At this incubation temperature, the tissues experienced minimal changes in morphology (Nakayama et al., 2010) and the lowest residual DNA values were obtained (Klak et al., 2021). A single decellularization process lasted 12 days. The decellularization or wash solution was changed 1-3 times per day. Avoiding recircularization and using fresh detergent solution results in increase decellularization efficacy (Schmitt et al., 2017). With each replacement of the rinsing solution, the material was cut with scissors. Tissue purification was performed with a neutral buffer - PBS without Ca^2+^ and Mg^2+^. On the 10th day nuclease-DNase I (0.001%) (Roche, Germany) dissolved in PBS with Ca^2+^ and Mg^2+^ for 8 h at 37 °C was used to eliminate the scattered DNA fragments after detergent treatment. After DNase digestion tissues were washed again with PBS solution for two more days with 3 h intervals per day.

**FIGURE 1:**
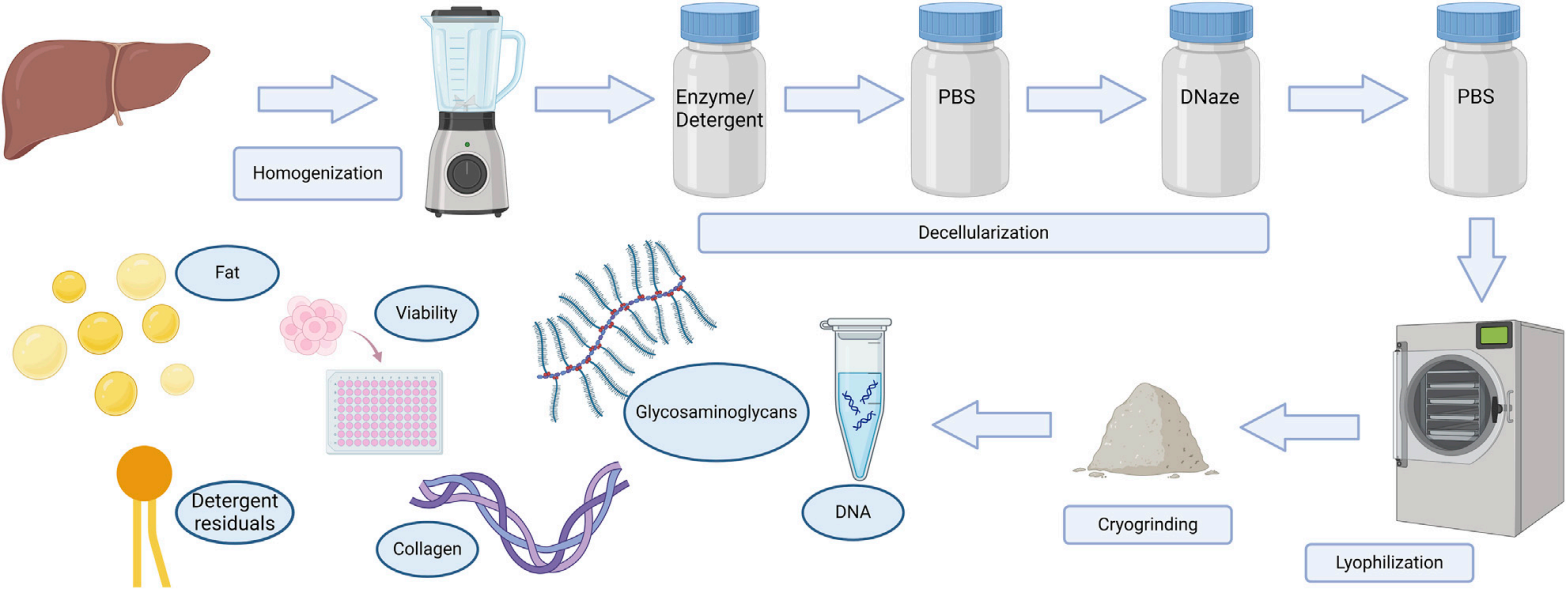
Schematic diagram of the decellularization process.

After the process was completed, both decellularized and fresh materials were prepared for lyophilization. A small amount of tissue was rubbed evenly along the walls of the chilled mortar. dECM was broken into small fragments and stored at −80 °C. The obtained lyophilizates were milled using a cryogenic mill so that the particle size did not exceed 100 nm. The grinding procedure included three 1 min cycles at a frequency of 15 beats per second. The lyophilizates were subjected to radiation sterilization. The general flowchart of the process is shown in Figure 1.

### 2.2 Biochemical analysis

DNA from tissues was isolated using Qiamp DNA Mini Kit (Qiagen, Germany) following the manufacturer’s instructions. Briefly, the samples were digested using Proteinase K and a digestion buffer. Samples were applied to columns for absorption of DNA. After steps of washes the DNA was precipitated and eluted. DNA samples were measured spectrophotometrically (Nanodrop, Thermo Scientific, US). Optical densities at 260 nm and 280 nm were used to estimate the purity and yield of nucleic acids. The average DNA content per mg of tissue, was determined. To separate and identify the size of the DNA fragments in dECM, electrophoresis was performed on a 1% agarose gel.

Soxhlet extraction was used for yield of lipids including: fats, waxes, sterols, fatty acids and others. 0.5 g of liver tissue (dry weight) were placed into cellulose thimbles and topped off with cottonwool. Lipids were extracted with 70 mL of n-hexane for 4 h, with a reflux cycle time of approximately 10 min. Weight content of the extracted lipids were determined in relation to the weight of the tested sample.

The sulfated glycosaminoglycan (sGAG) content of powdered fresh and decellularized organs was quantified using the Blyscan GAG Assay Kit (Biocolor, UK). In brief, 20–50 mg of dry tissue was weighed and placed in a 2 mL micro-centrifuge tube containing 1 mL of papain digestion buffer and incubated at 65 °C for 16 h, with occasional tube vortexing. Aliquots of each sample were mixed with 1,9-dimethyl-methylene blue (DMMB) dye and reagents from the GAG assay kit. The absorbance at 656 nm was measured using a microplate reader and compared to standards made from bovine tracheal chondroitin-4-sulfate to determine absolute sGAG content.

Acid soluble collagen quantification was performed using a commercial fluorescence test (Collagen Assay Kit, Sigma-Aldrich, US). Samples of the powdered matrix, dissolved in 0.5 M acetic acid and applied to a 96-well plate. Further steps were carried out according to the manufacturer’s instructions. Briefly, in the first step of this procedure, the acid soluble collagen in the sample is enzymatically digested into peptides. The N-terminal glycine-containing peptides then react with the dye to form a fluorescent complex. The fluorescence intensity of this product, measured at λex/em = 375/465 nm, is directly proportional to the collagen concentration in the sample.

Quantification of low ethoxylated non-ionic surfactant - Triton X-100 was conducted spectrophotometrically by the potassium salt of tetrabromophthalein ethyl ester (TBPE-K) (Klak et al., 2021). It consists in the formation of a colored complex of non-ionic surfactant with the TBPE-K reagent in a slightly alkaline environment and the measurement of the absorbance at 610 nm of the extracted TBPE-K-detergent complex with dichloromethane.

HPLC-CAD (high-performance liquid chromatography-Charged Aerosol Detector) analysis was performed on a UHPLC Ultimate 3000 HPLC system (Thermo Scientific, US) coupled to CAD. Separation was achieved on a Acclaim C8 120A column (150 × 4.6 mm; 3 μm) (Dionex) with the column temperature at 30 °C. Mobile phase A was 0.1% HCOOH and mobile phase B was MeOH. An izocratic elution was applied with the following program: 85 min (27% A, 78% B) at 1.2 mL/min. The injection volume was 20 μL. CAD parameters were set as follows: nebulization temperature: 25 °C; ion trap voltage: 20.4 V, gas pressure: 49.9–50.3 psi, charging current voltage: 2.4 V. Standard stock solutions of Tergitol 15.S.9 were prepared as follows: approximately 40 mg was accurately weighed into a 20 mL volumetric flask and dissolved using methanol (MeOH) as the diluent. The resulting Tergitol 15.S.9 solution had a concentration of 2 mg/mL. To prepare sample solutions approximately 200 mg of the test sample of the lyophilisate was weighed into a 2 mL centrifuge tube with a 0.45 μm PVDF filter, and 100 uL of diluent was added. The closed test tube was placed for 20 min of shaking and centrifugation at 15,000 rpm. After centrifuging the sample, the filtered solutions (approximately 30 μL) were analyzed. The total content of Tergitol 15.S.9 in the lyophilized samples was determined using the calibration curve method. The limit of detection (LOD) and limit of quantification (LOQ) were established.

The SDS Detection & Estimation Kit (G-Biosciences, US) was used to estimate SDS residues by colorimetric assessment of detergent residues in the wash solutions. A methylene blue solution was added to the detergent solutions to obtain a chromocomplex, which was fractionated by adding chloroform. The solution was vortexed and incubated at room temperature. The absorbance of the chloroform layer with suspended chromocomplex was measured at 600 nm.

### 2.3 Hydrogel preparation and cell viability

In total, seven hydrogel variants were prepared using three different enzymes (ficin, pepsin and collagenase). For each enzyme type, a corresponding control without enzyme addition was also prepared. The initial dECM concentration in all digestion mixtures was 5% (w/v). The % (w/w) concentration of enzyme refers to the enzyme-to-substrate ratio (E/S), i.e., enzyme mass per mass of dECM. All prepared hydrogel variants are listed in Table 2.

**TABLE 2:** Variants of prepared hydrogels based on selected dECM.

Biomaterial variant	dECM concentration in digestion mixtures [%w/v]	Pepsin concentration [%w/w]	Ficain concentration [%w/w]	Collagenase concentration [%w/w]	Final dECM concentration [%w/v]
HW5.P0	5	0			3.72
HW5.P1	5	1			4.14
HW5.P5	5	5			3.84
HW5.F0	5		0		5.00
HW5.F0.5	5		0.5		4.96
HW5.F1	5		1		4.93
HW5.C0	5			0	5.00
HW5.C1.37	5			0.137	5.00
HW5.C2.73	5			0.273	5.00
HW5.C5.47	5			0.547	5.01

Digestion with ficin (≥0.1 U*mg^-1^) (Sigma-Aldrich, US) was carried out at concentrations of 0, 0.5% and 1% (E/S) in demineralized water at 60 °C, 500 r*m^-1^ with magnetic stirring for 3 h. Although the enzymatic digestion was carried out in demineralized water, the use of a defined buffer system would provide better control of the reaction conditions.

dECM powder was digested in an acidic aqueous solution (0.1 M HCl, Sigma Aldrich, US) with pepsin (1 U*mg^-1^) (United States Pharmacopeia (USP) Reference Standard, Sigma Aldrich, US) at a concentration of 0, 1, 5% (E/S) in a water bath stirred magnetically at 500 r*m^-1^ for 48 h. The digested solution was neutralized to a physiological pH of 7.2–7.4 using a standard neutralization protocol consisting of 1 M NaOH (Fisher Scientific, US) and 10× PBS (Gibco, US) mixed at a 9:10 ratio. pH was monitored throughout the neutralization process using pH meter (Mettler Toledo).

Digestion with collagenase (≥0.90 U*mg^-1^) (Nordmark Biochemicals, Germany) at concentrations of 0, 0.137, 0.273% and 0.547% was carried out in Ringer’s solution with sodium bicarbonate (6.7 mM), HEPES (18.1 mM) (Serva, Germany), calcium chloride (7 mM). The Solution was neutralized to pH 7.2 with NaOH. The enzymatic reaction was carried out at 37 °C stirred magnetically at 1,000 r*m^-1^ for 15 min. The reaction was carried out in 50 mL bottles filled with 20 mL of solution. All digestion buffers were sterile-filtered (0.22 µm) and stored at 4 °C until use. A heating block was used to maintain the reaction at a constant temperature.

After neutralization and the addition of salt-containing buffers (e.g., NaOH, PBS), the final dECM concentration ranged from 3.72% to 5.01% (w/v) (Table 2).

To determine cell viability, obtained hydrogels were analyzed by MTT assay. A total of 10 variants were prepared (Table 2). The reference cell line L-929 cultured in DMEM supplemented with 10% fetal bovine serum (FBS, Gibco, UK) and 1% of antibiotics was used. Hydrogels were incubated for 24 h in complete culture medium at 37 °C (1 g/mL). Hydrogel extracts were applied to cells, which were incubated for 24, 48, 72 h. After incubation, the solutions were removed and 50 µL/well of MTT solution was added. Incubation was carried out under standard conditions for 2.5 h. Subsequently, the MTT solution was removed and 100 µL of DMSO was added. The plates were shaken until the crystals were completely dissolved. Absorbance was measured at 570 nm and 650 nm.

### 2.4 dECM applicapabilty in bioprinting

The procedure for developing a biomaterial of importance in 3D bioprinting technologies includes two parts. First, hydrogel preparation, followed by the formulation of the final biomaterial. A formulation was developed that ultimately resulted in the following concentrations of individual components: dECM in the form of a selected hydrogel (Table 2) at a concentration of 1% (w/v), GelMa (methacrylated gelatin, Polbionica S.A., Poland) 10% (w/v), HaMa (methacrylated hyaluronic acid, Polbionica S.A., Poland) 0.5% (w/v) with 0.2% (w/v) Lithium phenyl-2,4,6-trimethylbenzoylphosphinate (LAP 1.85 mg/mL; Polbionica S.A., Poland) was used as the photoinitiator. The material was stored in the refrigerator until use. GelMa (methacrylated gelatin, Polbionica S.A., Poland) 10% (w/v) and 0.25% LAP (LAP 2.50 mg/mL; Polbionica S.A., Poland) was used as a reference sample. The material with the developed formulation containing dECM from porcine liver was named dECM-based material, while the reference sample was named GelMa10 (10% GelMa and 0.25% LAP).

#### 2.4.1 Rheology

Rheological characterization of the materials was performed using an Anton Paar MCR 72 rheometer (Anton Paar, Austria) using a CP50 spindle. The parameters such as viscosity and complex modulus were determined. Due to its usefulness in the 3D bioprinting process, the biomaterial must be characterized by rheological properties such as storage modulus (G′), which refers to elastic properties and serves as a measure of elastic shape retention, and loss modulus (G″), which represents viscosity or the amount of energy dissipated in the sample (Schwab et al., 2020). For this purpose, the dynamic viscosity was measured at 25 °C temperature at a shear rate of 100/s. The complex modulus was also measured for a temperature of 20 °C at a constant frequency of 1 Hz and a variable amplitude of 0.1%–100%.

#### 2.4.2 Printability

The materials were printed using a BIOX Cellink printer (Cellink, Sweden). The printing speed, needle diameter and printing distance used in the test were 20 mm/s, 21 G (0.609 mm) or 25 G (0.437 mm) and 0.8 mm, respectively. The print was cross-linked with an external UV-Vis 365 nm lamp for 15 s at 13 W/cm^2^. A three-step evaluation procedure was used, using the Fiber Splicing Test printed as a pattern, the Fiber Collapse Test printed on a special platform and the Smoothness and Fiber Continuity during continuous printing (Naghieh and Chen, 2021).

#### 2.4.3 Fiber splicing test

In order to carry out the fiber splicing test, a g-code was designed. Layers were printed one after the other. The prepared print follows a 0°–90° pattern, which captures the 2D effect and increases the distance between fibers (FD). The distance between fibers was in the range of 1–5 mm with increments of 1 mm. The printing speed, needle diameter and printing distance used in the test were as follows: 20 mm/s, 0.609 mm or 0.437 mm and 0.8 mm. During the test, the material was dispensed at the appropriate range of pressures and temperatures. The printed construct was cross-linked with an external UV-Vis 405 nm lamp for 15 s at 13 W/cm^2^ Two parameters described by Equations 1, 2, [Disp-formula e2] were determined: the percentage of diffusion rate (spreading rate) (*Dfr*) and printability (*Pr*). The pore diffusion rate without material spreading is 0 (i.e., *A*_*t*_ = *A*_*a*_), and for an ideal reproduction of the model, the printability equals 1.

$$Dfr=\frac{At-Aa}{At\times 100\%}$$ (1)

$$\mathrm{Pr}=\frac{L2}{16\times Aa}$$ (2)

where *A*_*t*_ is the theoretical pore surface area, *A*_*a*_ is the actual surface area of the pore and *L* is the perimeter of the pore.

#### 2.4.4 Fiber collapse test

The deflection at the mid-span of the suspended fiber was analyzed to determine material collapse. For the experiment, a special platform consisting of seven pillars was designed and printed. The particular pillars were spaced from each other by 1, 2, 3, 4, 5 and 6 mm. The dimensions of the two corner pillars are 5 × 10 × 6 mm^3^, while the other five pillars are 2 × 10 × 6 mm^3^. Printing was performed at a speed of 20 mm/s with a 21 G needle (0.609 mm). The collapse area factor (*Cf*), defined as the percentage of the actual area after the deflection of the suspended fiber in relation to the theoretical area, was calculated using Equation 3:

$$Cf=\frac{Aca}{Act\times 100\%}$$ (3)

where *Aca* is the actual area under the curve and *Act* is the theoretical area under the curve.

If the material is too viscous and is unable to form a “bridge” between two pillars, the actual area is zero and the collapse factor equals 0. On the other hand, if the fiber does not collapse and forms a straight link between pillars, then *Act* = *Aca* and the factor is 100%.

#### 2.4.5 Smoothness and fiber continuity

The continuity of the fiber when printing 2–3 mL of the tested bioink was determined using a 0/1 system, where 0 means the fiber is broken and one means the fiber is continuous.

### 2.5 Statistical analysis

Quantitative results are reported as means ± standard deviation. Technical replicates were averaged, and all the statistical analyses were performed using three biological replicates. All grouped data was analyzed by one-way ANOVA with Tukey’s Post-Hoc testing. The equality of variances among groups was tested with a Levene test. The Shapiro–Wilk normality test was carried out and confirmed the normality distribution of the data. Welch’s t-test was used to compare the phase transition temperature, printability, diffusion rate and fiber bending between liver bioink and GelMa due to unequal variances in some samples. *p*-values below 0.05 were considered statistically significant.

## 3 Results

### 3.1 Decellularization

**FIGURE 2:**
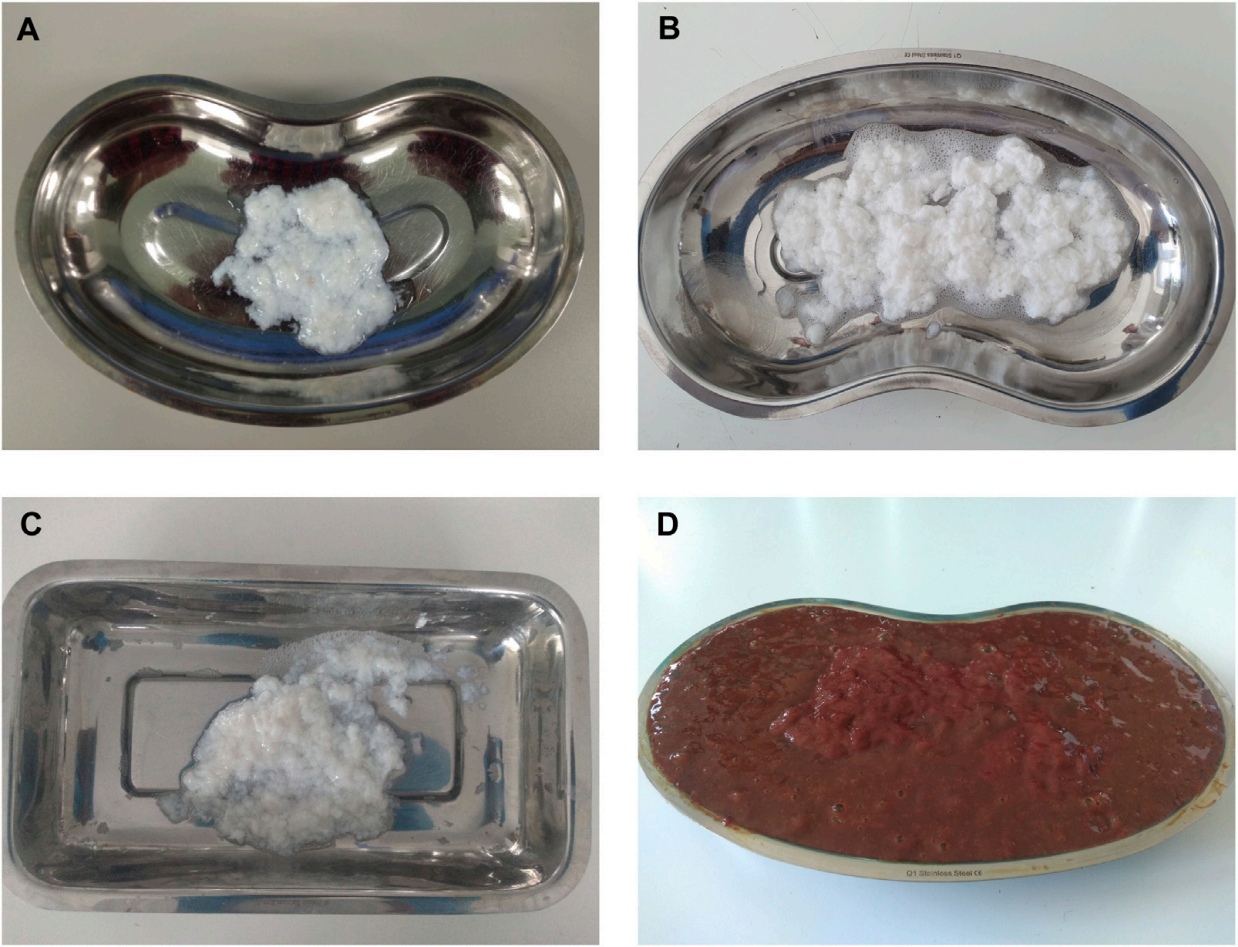
Differences between native and decellularised livers during the rinsing process. Liver tissue decellularized with: **(A)** protocol 9 (SDS/Tergitol + Trypsin), **(B)** Protocol 8 (Triton X-100/SDS + Trypsin), **(C)** Protocol 12 (Tergitol gradient + Trypsin), **(D)** blended native tissue.

13 decellularization protocols were compared. Examination demonstrated that color of tissue has changed from the initial red/pink color of the native liver tissue to almost or completely white (Figure 2). A little visible difference in color and amount of the tissue can be observed between some variants. Despite the similar visual effects of decellularization, the parameters of the final products obtained differed from each other.

### 3.2 Biochemical analysis

**FIGURE 3:**
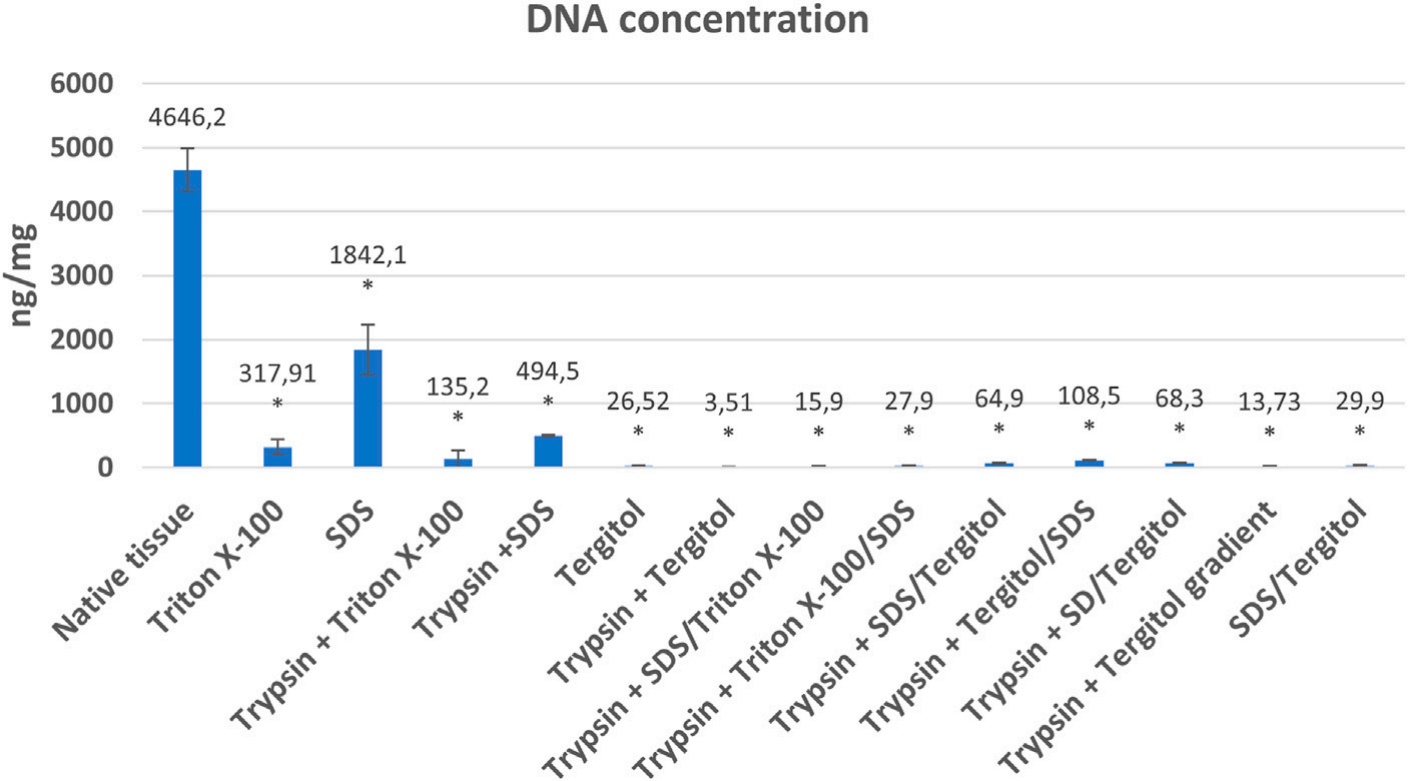
Mean DNA concentrations (ng/mg dry weight) and standard deviations in native tissue and powdered extracellular matrix obtained as a result of decellularization using individual methods. *indicates statistically significant difference compared to native tissue (*p* < 0.05).

DNA concentrations of powdered matrix obtained from each variant were significantly lower than in native tissue (Figure 3). Variants using: Tergitol (26.5 ug/mg ± 2.96), Tergitol in combination with enzymatic digestion (3.51 ng/mg ± 3.04), delularization in a Tergitol gradient (13.73 ng/mg ± 2.76), coupling of two detergents (SDS and Triton X-100) in two variants with pre-digestion in trypsin solution (SDS/Triton 15.9 ng/mg ± 1.69 and Triton/SDS 27.9 ng/mg ± 4.84) and the SDS/Tergitol variant (29.9 ng/mg ± 5.96) proved to be the most effective in removing residual DNA (*p* < 0.05). Double-stranded DNA decreased in decellularized tissues more than 99% compared to native livers which is an indirect proof of successful decellularization.

The use of Triton and SDS alone at 1% concentration was less effective in removing residual DNA. In the final product, it was 317.91 ng/mg ± 120.72 and 1842.08 ng/mg ± 392.96 for Triton and SDS, respectively. Variants in which the samples were subjected to enzymatic pre-digestion in most cases (Protocols: 1 vs. 3, 2 vs. 4 (*p* < 0.05) or 5 vs. 6) turned out to be more effective compared to variants using only detergent. Using the coupling of Triton X-100 and SDS, a clearly greater efficiency in DNA removal was demonstrated compared to decellularization using each detergent separately. A similar effect did not occur in the SDS/Tergitol and Tergitol/SDS experiments (Figure 3).

**FIGURE 4:**
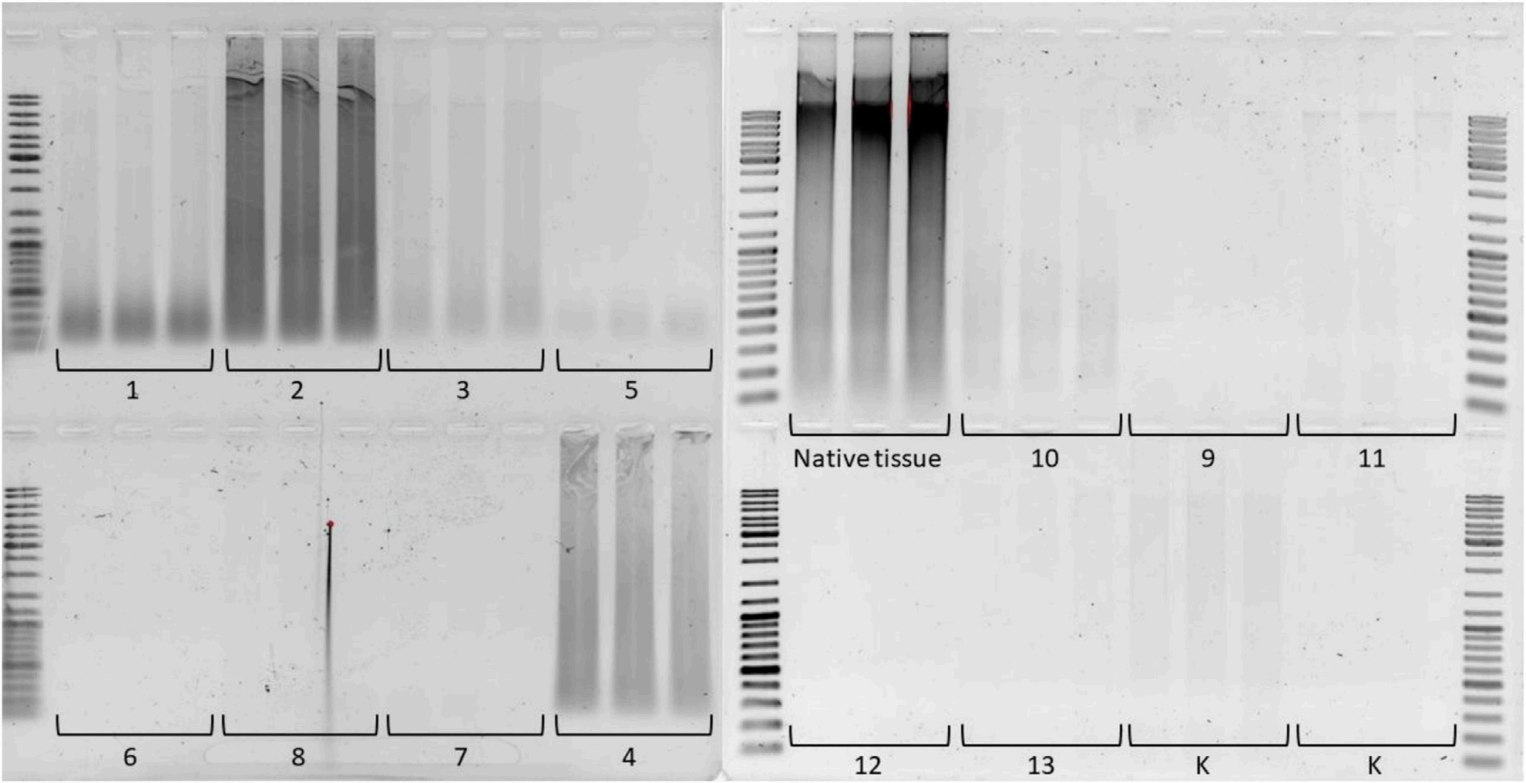
Electrophoresis of residual DNA isolated from liver dECM powder and native tissue on a 1% agarose gel. The molecular weight range is 10,000 to 100 base pairs. The numbers correspond to the decellularization protocol used.

In the case of the three variants shown at Figure 3: protocol 6 (Trypsin + Tergitol), protocols seven and 8 (Trypsin + 2 detergents (SDS/Triton and Triton/SDS)), as well as protocol 12 - Trypsin + Tergitol gradient, no DNA fragments were identified (Figure 4).

**FIGURE 5:**
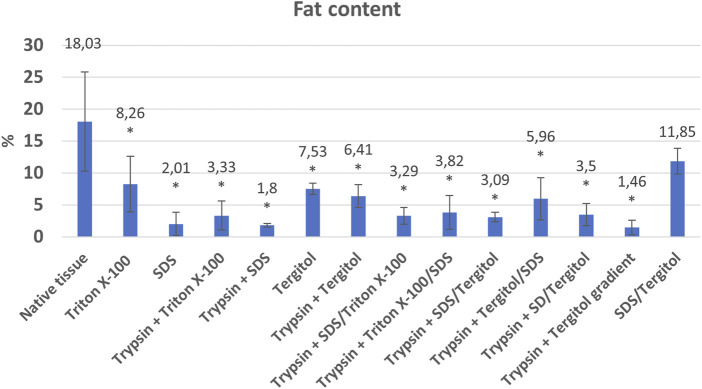
Percentage of fat in native tissue and powdered dECM after the decellularization process using individual methods *indicates statistically significant difference compared to native tissue (*p* < 0.05).

The fat content in the native organ averaged 18.03% ± 7.78 and showed big fluctuations. Most of the protocols used, allowed for a significant reduction of fat content in the final product compared to native tissue (Figure 4). The highest efficiency in fat elimination was demonstrated by the decellularization variant in the Tergitol gradient + NH_4_OH (1.46% ± 1.16, protocol 12). In the case of using a single detergent in a 1% concentration, the highest efficiency was shown by the ionic detergent – SDS (2.01% ± 1.82). This effect also occurred when SDS was coupled with a non-ionic detergent (Triton or Tergitol) and trypsin treatment. In both cases, a significant reduction in fat content was observed (Figure 5).

Non-ionic detergents such as Tergitol or Triton X-100 show lower lipids removal performance compared to SDS using a 1% detergent concentration, but still statistically significant with a fat content in the final product of 8.26% ± 4.37% and 7.53% ± 0.86 for Triton and Tergitol respectively.

**FIGURE 6:**
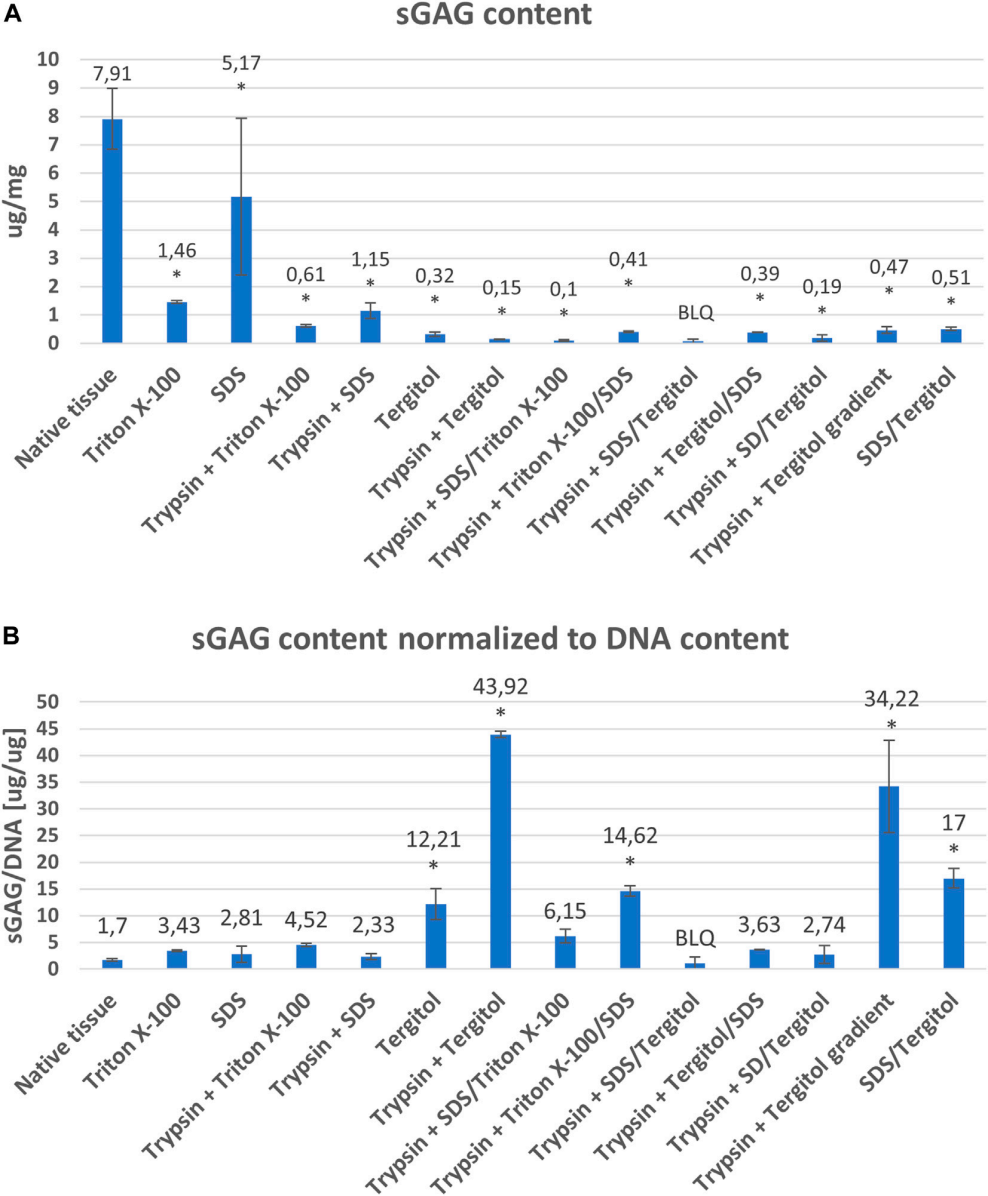
The content of glycosaminoglycans in the native tissue and powdered extracellular matrix obtained as a result of decellularization by individual methods in relation to dry weight (ngDNA/mg) **(A)** and in relation to DNA content (ugGAG/ugDNA) **(B)** *indicates statistically significant difference compared to native tissue (*p* < 0.05). BLQ–below limit of quantification.

A major challenge of the decellularization methods is the preservation of the liver-specific ECM components, which is essential for a successful repopulation of the decellularized liver. Unfortunately, sGAG content was lower in decellularized than control samples (*p* < 0.05). The highest score was obtained for the variant where decellularization was performed using SDS and was 5.17 ug/mg ± 2.76. The sGAG content was lower in samples where the decellularization process was more effective, as indicated by the DNA concentration results (<50 ng DNA/mg) (Figure 2). sGAG content was reduced from 7.91 ug/mg ± 1.07 to 0.51 ug/mg ± 0.05 in the case of the SDS/Tergitol variant (Protocol 13) and to even below the detection range in the case of the SDS/Tergitol + trypsin variant (Protocol 9) which is a decrease of over 90% (Figure 6A).

In contrast to the sGAG content per mg dry weight in decellularized tissue, sGAG accumulation normalized to DNA content was found to increase in most of the variants tested (Figure 6B). The greatest preservation of sGAGs in relation to the effectiveness of decellularization assessed on the basis of the content of residual DNA characterizes the decellularization carried out in the Tergitol gradient (protocol 12) and Tergitol + trypsin (protocol 6).

**FIGURE 7:**
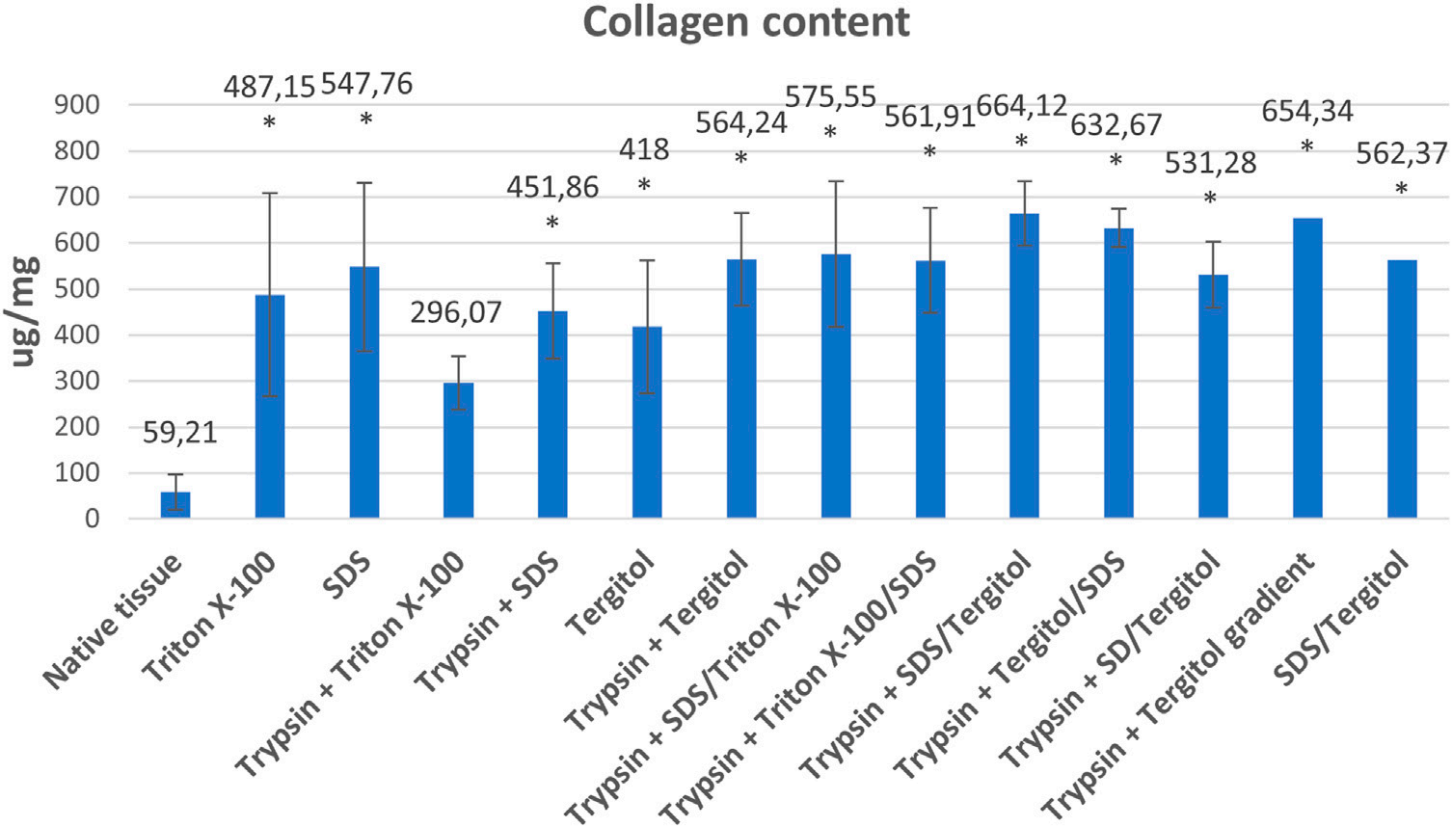
Collagen content in powdered native tissue and tissue decellularized by individual methods (ug/mg dry weight) *indicates statistically significant difference compared to native tissue (*p* < 0.05).

The collagen content is less affected by the decellularization method, and the preservation of this key ECM component was confirmed. An approximately 10-fold enrichment of the final material in collagen compared to the native tissue was observed (Figure 7). Values range from an average of 296.07 ug/mg ± 57.64 for tissue decellularized with Triton and trypsin (protocol 3) to 664.12 ug/mg ± 70.3 for tissue decellularized with the SDS/Tergitol + Trypsin (protocol 9) variant, but differences in collagen content were not statistically significant between decellularized tissues with the exception of protocol no. 3 (Triton X-100 + trypsin), where significantly less collagen was retained than in the others (*p* < 0.05).

Cleaning the tissues of detergent involves cyclic rinsing in a neutral solution. Residual detergent levels in decellularized porcine liver scaffolds were quantified. The obtained results are presented in Table 3.

**TABLE 3:** Residual detergent concentrations normalized to dry weight. n.a. - not applicable, n.e. - not examinated.

Protocol	Method	Average detergent concentration in ug per mg tissue			
		Triton X-100	SDS	Tergitol	SD
1	Triton X-100	<1	n.a	n.a	n.a
2	SDS	n.a	5.89	n.a	n.a
3	Triton X-100 + trypsin	<1	n.a	n.a	n.a
4	SDS + trypsin	n.a	<3.42	n.a	n.a
5	Tergitol	n.a	n.a	0.18	n.a
6	Tergitol + tryspin	n.a	n.a	0.05	n.a
7	SDS/Triton + trypsin	<1	<2.5	n.a	n.a
8	Triton/SDS + trypsin	<1	<2.5	n.a	n.a
9	SDS/Tergitol + trypsin	n.a	<2.5	0.03	n.a
10	Tergitol/SDS + trypsin	n.a	<2.5	0.02	n.a
11	SD/Tergitol + trypsin	n.a	n.a	0.01	n.e
12	Tergitol gradient + trypsin	n.a	<2.5	0.03	n.a
13	SDS/Tergitol	n.a	<2.5	0.66	n.a

The concentration of non-ionic detergents in the final products was low. The tests showed that the concentration of Triton X-100 after the process was below 1 μg/mg (Table 3).

Separation of Tergitol appears in three peaks in the chromatogram. The most specific Tergitol peak, well separated from impurities, was selected for quantitative analysis. The developed method demonstrated good linearity over the concentration range of 0.3–200 μg/mL, with an *R* ^2^ value of 1.0. The sensitivity analysis of the HPLC-CAD method showed the limit of quantification for Tergitol 15.S.9 at the level of approximately 5 ng/mg, the limit of detection was estimated at 2 ng/mg. The results of Tergitol content determination in the tested lyophilisate samples are presented in Table 3. The results, as in the case of Triton, were below 1 μg/mg.

In the case of the ionic detergent - SDS, its average content in the final product was almost 6 ug/mg, where only this compound was used as a decellularizing agent. Residual SDS content was also detected in the decellularized sample using SDS with pre-digestion in trypsin solution. In the remaining powdered matrix samples obtained using two detergents, SDS residues were below the detection limit (Table 3).

### 3.3 Hydrogel preparation and cell viability

**FIGURE 8:**
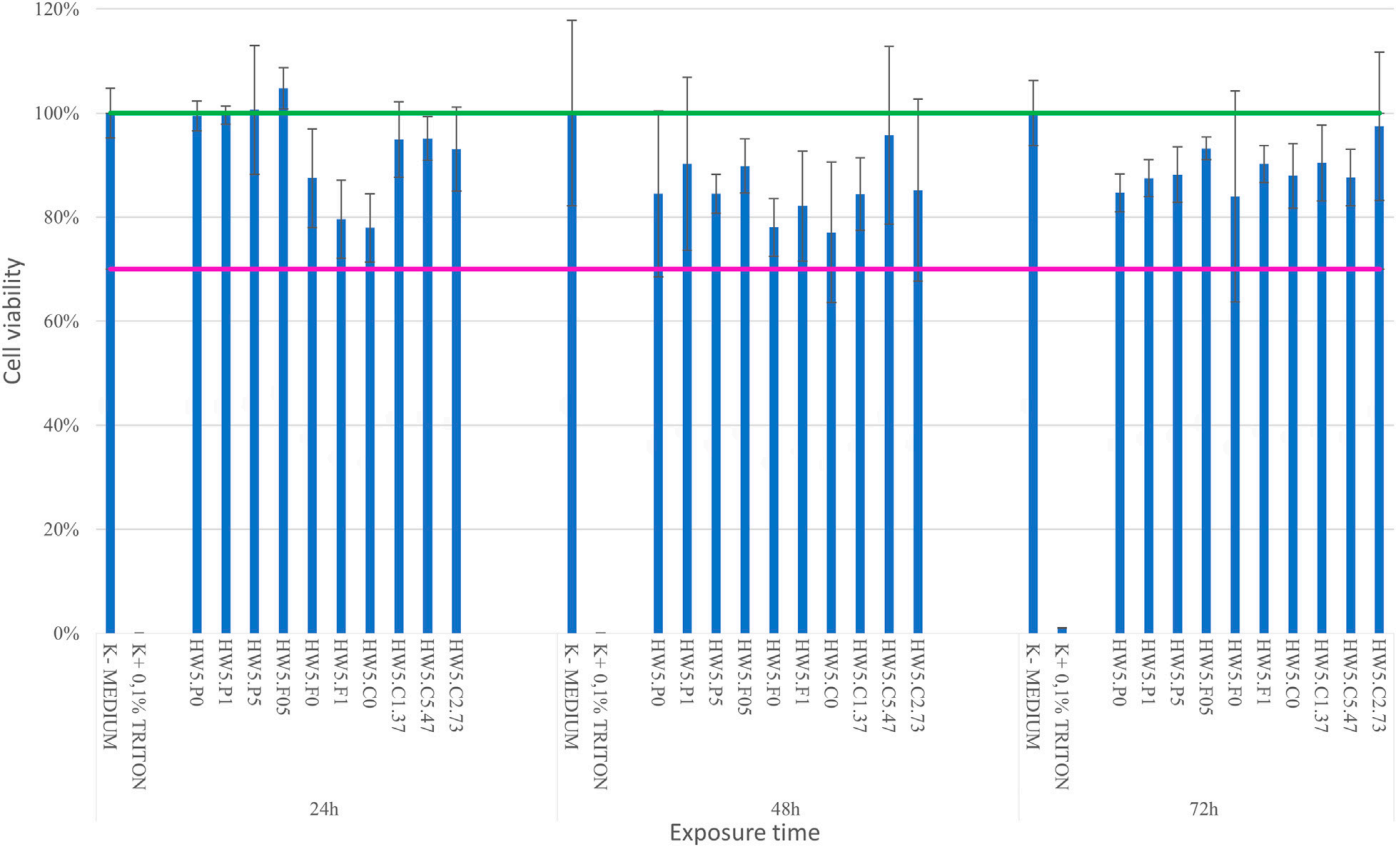
Cell viability after contact with hydrogels based on obtained dECM.

Cell viability was tested after contact with hydrogels produced from dECM prepared according to the protocol no. 12. 10 hydrogel variants were prepared. Exposure to hydrogel extracts was not toxic to the L-929 line. In each variant, cell viability was higher than 70% at all time points (24, 48, 72h) (Figure 8). For further studies, the hydrogel based on the HW5.P5 variant was used.

### 3.4 dECM applicapabilty in bioprinting

#### 3.4.1 Rheology

**FIGURE 9:**
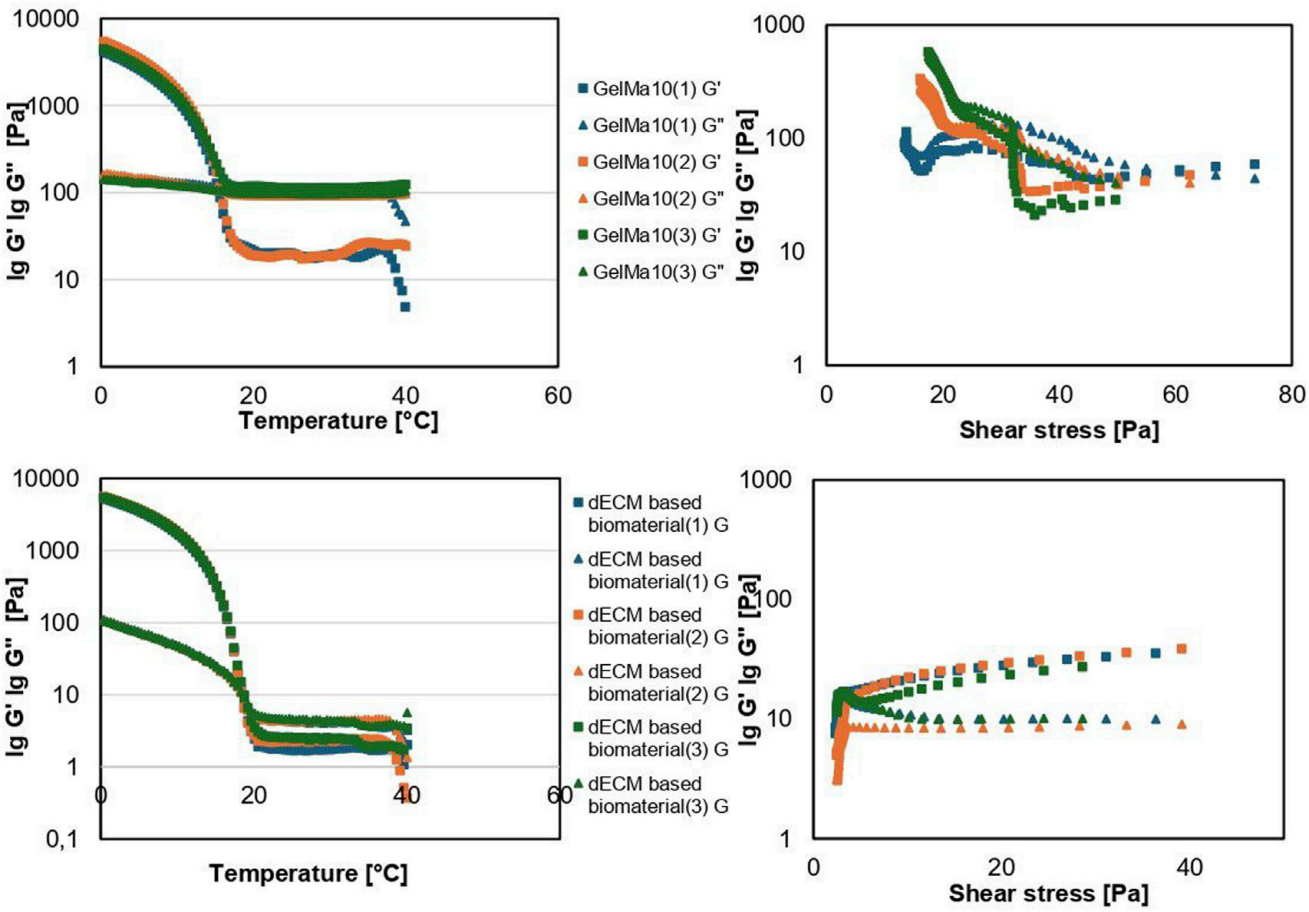
Rheological properties of the tested materials: gelation point and dependence of composite modulus on shear stress.

Figure 9 shows the results of rheological characterization of the obtained variants. The values of the storage modulus G’ in the studied amplitude range are 5–38.5 Pa, and the loss modulus G” is 3–17.5 Pa. A decrease in the value of the modulus of G” and an increase in the value of the modulus of G’ were observed with an increase in shear stress. The loss factor tanδ takes values below 1.0 in the range of shear stress greater than 5 Pa. Above this limit, the materials show an increase in fluidity while maintaining the gel structure. Compared to the reference sample – GelMa10, the range of G’ values measured under the same conditions was 21.04–578.38 Pa, and G” was 40.672 – 529.61 Pa. The viscosity of the tested material in 25 °C is close to 223 mPa*s, while for the reference material the average viscosity under the same conditions was 207 mPa*s. The study of the dependence of the composite modulus on temperature showed that the gelation temperature of all variants is in the range of 18.8 °C–19.25 °C and no significant differences were found in relation to the gelation temperature of GelMa10.

#### 3.4.2 Printability

**FIGURE 10:**
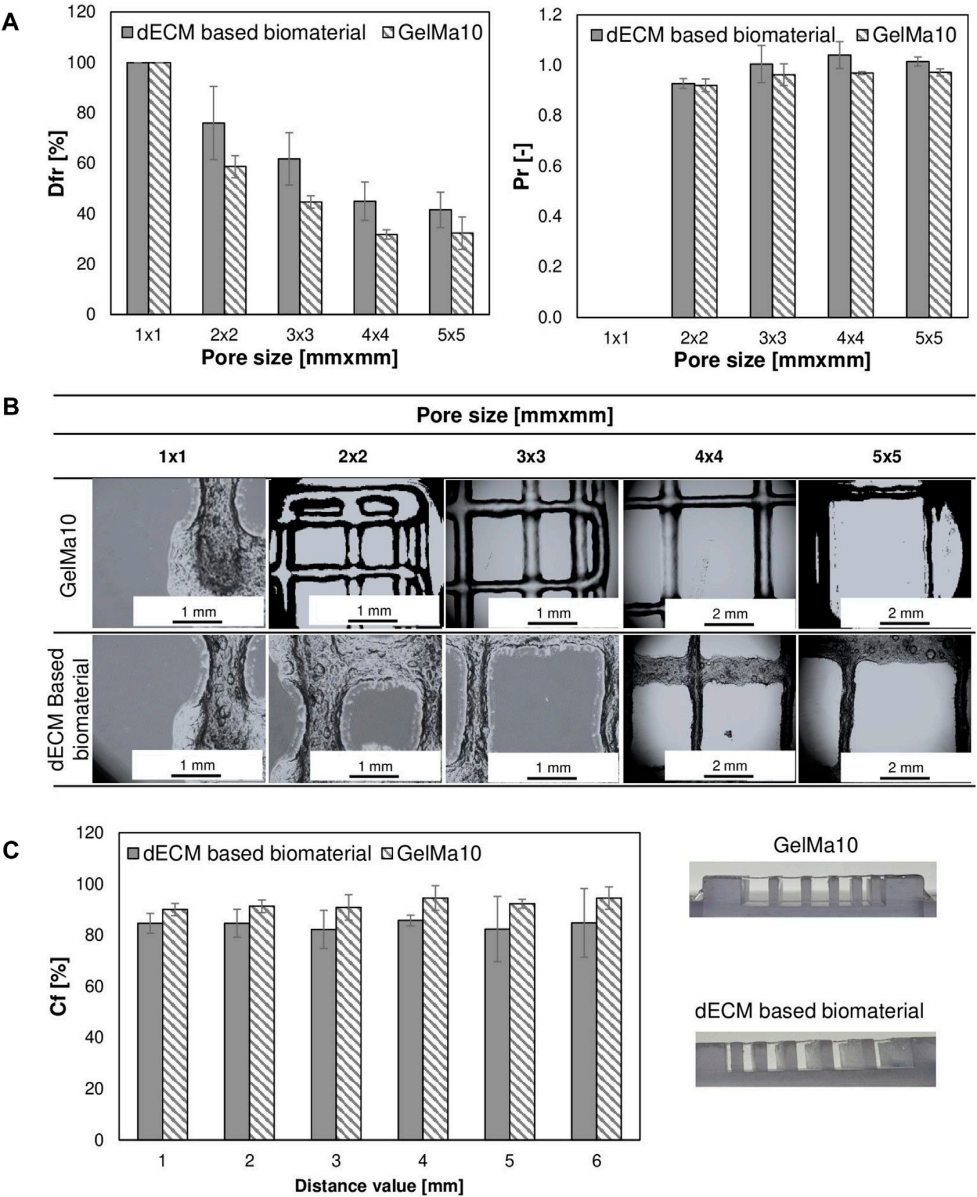
Analysis of the printability of tested materials **(A,B)** the test of splicing of printable fibers in the form of a pattern model **(C)** the test of fiber collapse.

The tested materials were printable under the following conditions: dECM-based material −19 °C, 44–48 kPa, 10 mm/s, GelMa10 °C–17 °C, 30–40 kPa, 10 mm/s using a 25 G nozzle, which are favorable conditions for the possibility of using them in bioprinting technology using living cells. Figure 10 shows the results of evaluating the printability of materials based on fiber continuity and smoothness. The materials exhibit good printability and print resolution, as the value of the diffusion coefficient decreases and printability increases with increasing pore size. Printability for all tested materials for pores larger or equal to 4 mm^2^ is above 80%. A continuous and smooth printed fiber was obtained for all tested materials on the platform. Printability and diffusion coefficient did not show any significant differences between the materials except for printability with a pore size of 5 mm^2^ where this value was slightly higher for the liver bioink.

## 4 Discussion

Normal cell physiology and function strongly depend on cell-cell and cell-extracellular matrix interactions in the 3D environment. Liver matrix has been shown to stimulate the expression of liver-specific functions, including albumin secretion, urea synthesis, upregulation of HNF4, cytochrome P450 and enzyme activity in ranges specific for this organ (Nakamura and Ijima, 2013; Brill et al., 2002; Navarro-Tableros et al., 2015). Findings prove that ECM scaffolds can promote stem cell hepatocyte differentiation in 3D culture and promotes faster cell maturation (Lorvellec et al., 2017) therefore, dECM could potentially be used in liver bioengineering (Lang et al., 2011). Hydrogels generated from decellularized livers are used as scaffolds for cell encapsulation, as an injectable material for *in vivo* healing, or as bioink for 3D bioprinting applications.

In the present study, we explored the use of a non-ionic detergents (Triton X-100, Tergitol), an anionic detergent (SDS, SD) and enzymatic agents (trypsin, DNase I) to decellularize porcine livers.

Surfactants act by disarranging the phospholipid cell membrane, thereby lysing cells (Parsi, 2015). Numerous examples of types of tissues and organs from both human and animal origin where SDS was successfully used in their decellularization can be found. Mouse pancreata were decellularized by perfusion using 0.5% SDS. Received matrix was free of cells and retained part of the pancreas ECM including the vasculature and its internal elastic basal lamina, the ducts with their basal membrane (Guruswamy Damodaran and Vermette, 2018). SDS has also been used in the decellularization of rat liver where the preservation of the original extracellular matrix was observed (Pan et al., 2014). The highest percentage of matrisome enrichment was obtained by SDS decellularization, but the number of identified matrisomal proteins was the lowest (Krasny et al., 2016). Other studies shown that SDS has the ability to denature proteins by disrupting protein-protein interactions damaging structural and signaling proteins. On the other hand, denaturation of proteins can be favorable, as it eliminates antigens which reduce the risk for immunogenic adverse effects.

Non-ionic detergents are usually preferred because of their more gentle effects on tissue structure and generally considered to be mild and relatively non-denaturing, as they break lipid–lipid interactions and lipid–protein interactions rather than protein–protein interactions (Seddon et al., 2004). Successful decellularization of porcine pancreas was achieved by immersing the tissue in 1% Triton X-100 solution (Klak et al., 2021). Decellularization with Triton X-100 allowed the preservation of two proteoglycans: lumican (LUM) and decorin (PGS2) (Krasny et al., 2016). Triton X-100–treated annulus fibrosus retained the major ECM components after thorough cell removal (Xu et al., 2014), but there are reports that Triton alter some properties of ECM (Mendoza-Novelo et al., 2011).

Crapo et al. proposed minimal criteria for elimination of residual DNA and nuclear material that, according to the authors, would be sufficient to validate decellularisation. The proposed criteria suggested that the decellularised ECM should contain less than 50 ng of dsDNA per mg of ECM (dry weight) with fragments smaller than 200 bp (Crapo et al., 2011). In the present study, DNA quantification revealed a significant reduction in DNA content between the fresh liver and the liver subjected to any decellularization treatment. The results of DNA quantitation demonstrated that non-ionic detergents were more effective in reducing the amount of DNA in decellularized scaffolds compared to SDS (Figure 3). Tergitol proved to be the most effective. Triton X-100 was also a more effective detergent for decellularization and DNA removal of human kidneys compared with SDS (Shahraki et al., 2022). In other studies, both perfusion decellularization procedures, which employed SDS with and without a subsequent DNase incubation, resulted in a strong decline of the liver DNA amount which corresponds to about 3% and 1% of the normal liver. However, the authors failed to reach the level of DNA proposed by Crapo (Bühler et al., 2015), but DNA removal to levels below 50 ng/mg and 100 bp is also achievable with SDS (Croce et al., 2022). The majority of DNA is removed from ECM devices, but that small amounts remained in most tested materials (Gilbert et al., 2009).

The use of trypsin and EDTA in our research allowed for slightly more efficient DNA removal, but it has been also reported that the use of trypsin for decellularization results in damage to the ECM components (Schenke-Layland et al., 2003). It was suggested that 1% trypsin should not be used longer than 24 h to prevent collagen damage in porcine dermis (Prasertsung et al., 2008). It is worth noting that trypsin activity decreases over time through inhibition by natural protease inhibitors released from lysed cells. In another study addition of EDTA further reduced the DNA content compared with detergent treatment alone (Maghsoudlou et al., 2016). Successful liver decellularization was carried out by retrograde perfusion using combination of trypsin, EGTA and Triton X-100 (Soto-Gutierrez et al., 2011).

The ideal decellularized ECM should preserve collagen and GAG content close to that of natural. GAGs are long highly negatively charged heteropolysaccharides that contain repeating disaccharides. Sulphated GAGs are an important component of the ECM, through their involvement in growth factor sequestration and presentation (Mullen et al., 2010). GAG side chains participating in several cell functions, such as cell signaling, migration, differentiation, adhesion and viral infections (Morla, 2019).

Most of the GAGs was removed during kidney decellularization process by SDS (Keshvari et al., 2021) or stomach by SD (Zambaiti et al., 2019). Decellularization protocol based on 4% Triton affected the GAG content of native liver (Nakamura and Ijima, 2013). GAG content of both detergent-enzymatic with EDTA and without EDTA treatment scaffolds was significantly reduced compared with fresh tissue (Maghsoudlou et al., 2016). The sGAG content also decreased significantly after all protocols treatments compared with the normal sheep uterus tissue (Tiemann et al., 2020). >60% of the GAG content was preserved in pancreas by decellularization with Triton and SDS, but in smaller concentrations (Hashemi et al., 2018). It appears that livers decellularized with Triton retained more GAG than decellularized SDS (Willemse et al., 2020). In contrast other study showed, GAGs levels increased from 0.67 mg/mg dry weight ECM to 3.22 and 5.3 mg/mg dry weight in SDS- and SDS/DNase-treated porcine livers (Bühler et al., 2015). The reason for this is currently unclear but might be due to use of not specific DMMB which is widely used in tests for the assessment of sGAG in decellularized matrices. Presence of SDS may cause positive interferences with DMMB. This may explain why the quantification of GAG content in liver decellularized by perfusion was found to be higher than in native samples before decellularization (Bühler et al., 2015), and also why, in our study, the amount of GAGs appears to be higher after SDS decellularization (Figure 6A). Research suggests that the loss of GAG is related to the detergent-enzymatic process of decellularization, but this reduction may be compensated for by the addition of soluble sGAGs before recellularization.

Collagen is comprised of twenty-nine different types (I-XXIX) (Gordon and Hahn, 2010; Söderhäll et al., 2007). Collagen content normalized to the total protein ranges approximately from 0.1% to 50% in mice depending on the type of tissue (Tarnutzer et al., 2023). Cells have the ability to bind to the collagens via receptors (Heino, 2007). It is known that ECM proteins can influence proliferation of HepG2 cells. For example, collagen I promotes HepG2 proliferation via regulation of the Integrin β1/FAK signaling pathways (Zheng et al., 2017). MIN6 beta-cells cultured in gels containing collagen type IV or laminin secreted more insulin in response to glucose stimulation than beta-cells alone (Weber et al., 2008). Interstitial collagens I, III, V, and VI contain low-affinity binding sites for HGF (hepatocyte growth factor) in the ECM, suggesting a potential role in modulating HGF availability (Schuppan et al., 1998). In this study significant enrichment of the powdered decellularized matrix in collagen relative to the native tissue was observed (Figure 7). Other studies have produced mixed results. After the perfusion of SDS or SDS/DNase collagen levels indicating a preservation of 57% and 68% respectively (Bühler et al., 2015) or preservation of collagen type I during the decellularization process (Uygun et al., 2010). Collagen quantification of the scaffolds obtained using SD/Dnase (Maghsoudlou et al., 2016), SDS or Triton X-100 (Ren et al., 2013) treatment demonstrated no significant changes when compared to fresh tissue, but with the addition of EDTA scaffolds had increased collagen content (Maghsoudlou et al., 2016), other studies indicate an increase collagen content scaffold obtained by perfusion with Triton and SDS (Mazza et al., 2015; Willemse et al., 2020). An increase in the relative content of collagen, is due to the fact that, the ratio between the different ECM components changes in respect to the overall tissue weight, by removing cells.

Too high fat content can negatively affect the rheological properties of bioinks, which makes bioprinting of constructs difficult. The implication of remaining fat droplets for recellularization is not yet clear. Sterilization based on gamma radiation may affect the formation of cell-damaging compounds in the scaffolds. In one of the studies slices were prepared from bone cores and were transferred on osteoblast-like cell layers (Saos-2). The slices with lipids did not induce cell death. However, cell death was dramatically increased around the gamma-irradiated slices where peroxidated lipids appeared from two to 3-fold higher. Defatted slices which had been sterilized by gamma radiations or UV did not induce cell death (Moreau et al., 2000). Therefore, the amount of fat after decellularization may be relevant. It is worth noting that differences in fat content in scaffolds may result from its variable amount in native tissue (Figure 5).

Most decellularized scaffolds are screened for the presence of ECM components; however, in the context of effective recellularization and medical applications, measuring the residual detergent concentration is also essential.

Despite the long washing protocol, SDS residues were detected in our studies (Tab. 3). SDS is cytotoxic, and therefore, it must be thoroughly washed after the decellularisation process. Triton X-100 was able to elide the residual SDS which ensured the scaffolds were not cytotoxic to cells (Wang et al., 2015). Both PBS and bovine serum albumin perfusion showed weaker effects on the elimination of residual SDS than Triton. Decellularisation of small intestine submucosa showed that a protocol using SDS/Triton X-100 surfactants yielded reduced metabolic activity due to the cytotoxic effect of residual agents (Syed et al., 2014). On the other hand, some residual detergent is tolerated by the cells. Detergent concentration of <50 mg/L in the wash solution did not influence the receptiveness of the matrix to reseeding with endothelial cells (Cebotari et al., 2010). Cytotoxicity test showed that decellularized trachea tissues did not induce cytotoxicity in Wharton’s jelly mesenchymal stromal cells (Dimou et al., 2019). Some researchers report that alterations in ECM biochemistry or structure from SDS treatment are more responsible for the low cell re-population observed in SDS decellularized tissues than residual SDS (Gratzer et al., 2006) and lower cytotoxic threshold for SDS is approximately 10 ug/mg dry weight (Keane et al., 2015).

Due to the greater negative impact on tissues and greater difficulties in removing ionic surfactants using typical solutions like PBS, non-ionic agents are preferred. Triton X-100-treated scaffolds reseeded with hepatocytes were superior in supporting liver-specific functions, including: albumin secretion, urea synthesis, ammonia elimination and mRNA expression levels of drug metabolism enzymes to a greater extent than SDS-treated scaffolds (Ren et al., 2013). On the other hand perfusion with Triton X-100 alone was not sufficient for decellularization and SDS was required for complete nuclear removal (Shupe et al., 2010).

We also used another non-ionic detergent, a secondary ethoxylated alcohol - Tergitol, in our research. So far, it has been used in decellularization of the porcine aortic valves (Faggioli et a., 2022), porcine pulmonary valve (Tondato et al., 2023), porcine pericardia (Todesco et al., 2022) and small intestinal submucosa (Casarin et a., 2022). Tergitol is less toxic and highly degradable (Li and Chen, 2009). In our method using a Tergitol gradient, dECM were not cytotoxic to L929 cell lines but extended culture periods will be required in future research to investigate long-term cell-material interactions and the bioink’s performance in tissue engineering applications. Still, the testing of detergent residues in scaffolds is important. Tergitol, and its degradation product NP-9 affect the physiology of *Caenorhabditis elegans* and modulate gene expression related to ROS production, cellular stress and metabolism of xenobiotics (De la Parra-Guerra and Olivero-Verbel, 2020). Complete elimination of detergent residues is unlikely, making it necessary to determine their actual levels in the scaffolds. The results of the current study demonstrated that HPLC method with CAD can be a straightforward feasible method for separation and quantification of residual Tergitol present in scaffolds. CAD is based on nebulization of the eluent, generating droplets. After evaporation of these droplets in the drying tube, analyte particles are formed, increasing the likelihood of detecting all components, including non-UV-absorbing compounds. The method is characterized by greater sensitivity and selectivity than spectrometric methods. In recent years a HPLC method coupled to CAD was developed, for example, for simultaneous separation and quantification of the nine impurities (Huang et al., 2023), residual sodium deoxycholate in the bulk of hepatitis A and pneumococcal vaccine (Shao et al., 2023), compositional changes in polysorbates upon changes in pH (Evers et al., 2020).

The obtained rheological properties confirm the suitability of the tested material for use in extrusion bioprinting technology. The phase transition point is at or above that of the reference sample. The tested materials were printable under defined conditions, with printability comparable to the reference material, which is widely used for 3D model printing in the literature. Based on the compiled results, we conclude that the tested material is less compact than the reference sample, which may be advantageous for biomedical applications involving cell-laden tissue model printing. For example, Mao et al. (2020) showed that supplementing 10% GelMA with liver-derived dECM significantly improved cell viability without compromising the mechanical and rheological properties of the material, highlighting the beneficial impact of dECM on cell-supportive environments. Similar findings were reported by Kim et al. (2023), who developed a gelatin-based composite bioink incorporating liver dECM. This material allowed for precise 3D printing and supported the formation of highly functional liver tissues. Moreover, Shi et al. (2024) emphasized the importance of decellularization protocols, showing that different detergents used during the dECM preparation process directly affect the biological and mechanical properties of the resulting bioink.

Cell viability alone does not provide a complete picture of bioink performance, and that functional evaluation including assessments of proliferation capacity and tissue-specific marker expression is essential for a comprehensive analysis such as: key hepatic markers (e.g., albumin, CYP enzymes, and urea cycle enzymes), particularly in hepatocyte cultures.

In conclusion, decellularized livers obtained using our method with Tergitol using for the first time to our knowledge in liver decellularization demonstrated competitive performance exhibited residual DNA content below 50 ng mg^-1^ dry ECM, with DNA fragment lengths under 200 bp meeting commonly accepted decellularization criteria. Collagen and some of sGAGs retention was maintained relative to native liver tissue (Figures 6, 7). The rheological profile of the bioink, including dominant elastic behavior, shear-thinning flow, and rapid viscosity recovery, directly supports its gelation capacity and high print fidelity. Additionally, we developed a method for detecting Tergitol in decellularized matrices with LOQ of 5 ug/mg, excellent linearity across the relevant concentration range, and high recovery of the analyte. Liver decellularization remains a challenge due to the complex structure and high cellularity of this organ. Species differences, such as the presence of collagen septa in porcine liver, influence ECM composition and may compromise its functionality in the context of human cells. This requires optimized protocols, particularly with respect to preserving soluble ECM components and minimizing the risk of immunogenicity (e.g., the presence of α-gal, MHC, Porcine Endogenous Retroviruses - PERV). Successful translation of porcine materials for human applications should include validation of function in human cells (albumin, urea, CYPs) and assessment of *in vivo* biocompatibility. These results suggest that ECM can support liver function, but its species origin is crucial. Further investigations are needed to optimize decellularization protocols to standardize procedures to establish minimal criteria for evaluating decellularization success.

## Data Availability

The raw data supporting the conclusions of this article will be made available by the authors, without undue reservation.
